# Durability and Microstructure of Fly Ash/Silica Fume-Modified Geopolymer Concrete with Inorganic Aluminosilicate Polymer Gels Under Freeze–Thaw Cycles and Single-Side Salt Erosion

**DOI:** 10.3390/polym18121514

**Published:** 2026-06-17

**Authors:** Jianghuai Zhan, Lepeng Huang, Chao Li, Xuanyi Xue, Kai Xu, Jilin Song, Shuai Li, Jianmin Hua

**Affiliations:** 1 School of Civil Engineering, Chongqing University, Chongqing 400045, China; 2 State Key Laboratory of Safety and Resilience of Civil Engineering in Mountain Area, Chongqing 400045, China; 3 Support and Service Center of China Academy of Engineering Physics, Mianyang 621999, China; 4 China Construction Third Engineering Bureau Group Co., Ltd., Wuhan 430064, China; 5 Department of Civil Engineering, The University of Hong Kong, Pokfulam Road, Hong Kong, China

**Keywords:** geopolymer concrete, fly ash, silica fume, freeze–thaw cycles, single-side salt freeze–thaw cycle erosion, chloride ion transport, microstructure

## Abstract

Geopolymer concrete contains inorganic aluminosilicate polymer gels formed through the activation of industrial solid wastes. This study investigated the effects of fly ash (FA) and silica fume (SF) on the durability and microstructure of geopolymer concrete exposed to freeze–thaw cycles and single-side salt erosion. Five mixtures were prepared using Baioheng geopolymer cement, with FA replacement levels of 15% and 25% and SF replacement levels of 3% and 5%. Mechanical tests, freeze–thaw tests, single-side salt-freezing tests, SEM-EDS, XRD, and CT analysis were conducted to evaluate the relationship between macroscopic performance and inorganic polymer gel structure. The results showed that 25% FA reduced compressive strength and freeze–thaw resistance, mainly due to insufficient reaction products and increased defect connectivity. In contrast, 3% SF improved the 56 d compressive strength by 13.24%, maintained the relative dynamic elastic modulus at 86.64% after 100 freeze–thaw cycles, and limited the mass loss to 0.72%. SEM-EDS and XRD results indicated that appropriate SF addition increased the Si/Al ratio and promoted the formation of C-(A)-S-H/N-A-S-H-related gel products, leading to a denser inorganic polymer matrix. However, excessive SF weakened the improvement effect, possibly due to local heterogeneity and dispersion difficulty. These results indicate that controlling the composition and spatial distribution of inorganic aluminosilicate polymer gels is essential for improving the salt-frost durability of geopolymer concrete.

## 1. Introduction

Conventional Portland cement was the most widely used man-made construction material in the world. Its production was accompanied by high energy consumption and high carbon emissions. Each ton of cement emits approximately 0.8 to 1.0 tons of CO_2_. The cement industry contributed about 7% of global anthropogenic CO_2_ emissions. This had become a major constraint on achieving the “dual carbon” goals [1,2,3]. A new type of silica-alumina-based low-carbon cement used industrial solid wastes as its main raw materials. These wastes included fly ash, slag, and steel slag. Through mechanical activation and chemical excitation, this cement significantly reduced or even eliminated limestone calcination. It thereby reduced carbon emissions at the source [4]. However, systematic research was still lacking on the synergistic effects between this type of low-carbon cement and supplementary cementitious materials such as fly ash (FA) and silica fume (SF). Its long-term performance evolution mechanism under complex environments was also not well understood. This knowledge gap severely restricted the standardization and engineering application of these materials. From the perspective of polymer science, geopolymer concrete is not merely a cement-based composite, but a material containing inorganic aluminosilicate polymer gels. Its performance is closely related to the formation, composition, and spatial continuity of N-A-S-H and C-(A)-S-H type gel networks. Therefore, the key scientific problem of this study is to clarify how FA and SF regulate the inorganic polymer gel structure and how this regulation affects freeze–thaw resistance, chloride transport, and salt-frost damage.

FA had a pozzolanic effect. It could react with calcium hydroxide, a hydration product, to form additional C-S-H gel. This improved the long-term strength and durability of concrete. However, its early-age activity was low [5]. SF had high pozzolanic activity and a micro-aggregate filling effect. It could refine the pore structure and optimize the interfacial transition zone. It significantly enhanced the strength and chloride ion penetration resistance of concrete [6,7]. Research showed that the incorporation of SF could reduce the chloride ion migration coefficient by more than 50% [8]. The combined incorporation of FA and SF further refined the pore structure. It also reduced permeability and delayed the degradation process [9]. The incorporation of SF significantly improved the freeze–thaw resistance and salt erosion resistance of concrete [10,11].

Recent studies on geopolymer and alkali-activated systems have mainly focused on mechanical properties, durability, and pore structure modification by mineral admixtures. However, fewer studies have discussed these changes from the perspective of inorganic polymer gel structure and its relationship with salt-frost durability. A summary of representative recent studies is provided in Table 1.

In cold coastal regions and salt-frost areas, concrete structures often faced the coupled effect of freeze–thaw cycles and chloride salt erosion. This was one of the main factors leading to durability deterioration [8,12,13]. Freeze–thaw cycles could increase the chloride ion diffusion coefficient by 2 to 5 times. The compressive strength loss rate could reach 20% to 50% [14]. Research indicated that chloride ion binding mainly occurred in two forms: chemical binding and physical adsorption [15,16]. Chemical binding mainly involved the reaction of AFm phase [17] and hydrated calcium aluminate [18] with chloride ions. This reaction formed Friedel’s salt and Kuzel’s salt [19]. Physical adsorption mainly depended on C-S-H [20]. Chloride ion binding could significantly reduce the free chloride ion content in the pore solution. This, in turn, affected the transport behavior of chloride ions. In coastal salt-frost environments, the free chloride ion content also influenced the freezing temperature and crystallization pressure of the pore solution. This led to differential frost heave forces at different erosion depths, which further aggravated the damage. Single-side salt freeze–thaw cycle caused repeated freezing and thawing of pore water. This promoted chloride ion transport [21]. Du et al. [22] investigated the transport and binding behavior of chloride ions in concrete under single-side salt freeze–thaw cycle. They found that the coupling effect of freeze–thaw cycles and chloride ion erosion was most significant at a chloride ion concentration of 10%. After 100 single-side salt freeze–thaw cycle, the compressive strength of concrete decreased by 14.2% compared with that under pure water freeze–thaw. The mass loss increased by 383%. Jin et al. [14] monitored the diffusion behavior of free chloride ions in concrete after freeze–thaw cycles using embedded sensors. They found that the degree of freeze–thaw damage had an exponential relationship with the chloride ion diffusion coefficient. The greater the damage degree was, the faster the chloride ion diffusion coefficient increased. Xia et al. [23] investigated the damage characteristics of hybrid fiber-reinforced concrete under freeze–thaw cycles and composite salt erosion. They found that the incorporation of SF significantly improved the freeze–thaw resistance and salt erosion resistance of concrete. Li et al. [10] investigated the mechanical properties of hybrid fiber-reinforced recycled rubber concrete under freeze–thaw cycles. They found that hybrid fibers reduced the compressive strength loss rate to 9.3% after 100 freeze–thaw cycles. Some scholars investigated the durability of structural materials after disasters [24,25,26,27]. However, all of the above studies were conducted on OPC systems. Systematic research was still lacking on the long-term performance evolution of new low-carbon cement-based concrete under coupled salt-frost environments. In addition, the regulation mechanisms of SCMs on the microstructure (e.g., Ca/Si ratio, degree of C-S-H polymerization, pore structure, etc.) had been extensively studied in OPC systems [28,29]. However, these mechanisms remained unclear in new silica-alumina-based low-carbon cement.

Based on this, the present study used low-carbon cement as the cementitious material. Different dosages of FA (15% and 25%) and different dosages of SF (3% and 5%) were designed. Geopolymer concrete specimens were prepared accordingly. Systematic macro-mechanical performance tests were conducted. These included compressive strength tests, freeze–thaw cycle tests, and single-side salt freeze–thaw cycle tests. Microstructural characterization was also performed using SEM-EDS, XRD, and CT. The effects of FA and SF on the mechanical properties, freeze–thaw resistance, salt-frost scaling resistance, and chloride ion transport behavior of geopolymer concrete were investigated. The microstructural evolution mechanisms were revealed. The research findings could provide a theoretical basis and technical support for the engineering application of geopolymer concrete in coastal salt-frost environments. These findings are particularly relevant to concrete structures in cold coastal regions, splash zones, tidal zones, and deicing-salt environments, where chloride ingress and freeze–thaw damage occur simultaneously from exposed surfaces.

**Table 1:** Recent studies on mineral admixture-modified geopolymer and low-carbon cementitious systems.

Reference	Material System	Main Focus	Key Findings	Research Gap
Liu et al. [6]	Alkali-activated concrete	Sodium chloride attack and freeze–thaw cycles	Alkali dosage and silicate modulus affected the deterioration behavior under chloride attack and freeze–thaw cycles.	The relationship between inorganic polymer gel structure and salt-frost resistance was not fully discussed.
Dong et al. [7]	Concrete under coupled actions	Freeze–thaw cycles, loading, and chloride transport	Freeze–thaw damage accelerated chloride transport and affected durability degradation.	The study mainly focused on transport behavior rather than inorganic polymer network evolution.
Wang et al. [8]	FA/SF-modified concrete	Chloride penetration under combined freeze–thaw and chloride attack	SF improved chloride penetration resistance and enhanced durability.	The role of aluminosilicate gel composition and polymer gel continuity was not emphasized.
Lv et al. [9]	High-ferrite cement with FA and SF	Abrasion resistance and microstructure	SF showed a stronger improvement effect than FA on material resistance and microstructure.	The effect of FA/SF on geopolymer gel structure was not the main focus.
Du et al. [22]	Concrete under single-side salt-freezing cycles	Chloride transport and binding behavior	Single-side salt-freezing cycles significantly affected chloride ion transport and binding.	The discussion was mainly based on OPC chemistry rather than geopolymer or alkali-activated gel mechanisms.
Xia et al. [23]	Hybrid fiber-reinforced concrete	Freeze–thaw cycles and compound salt attack	SF improved freeze–thaw resistance and salt erosion resistance.	The relationship between polymer-related reaction products and durability was not clarified.
This study	Baioheng geopolymer cement modified with FA and SF	Inorganic aluminosilicate polymer gel structure and salt-frost durability	FA and SF changed Si/Al ratio, Ca/Si ratio, reaction products, pore defects, chloride transport, and freeze–thaw resistance.	This study links mineral admixture effects with inorganic aluminosilicate polymer gel evolution and salt-frost durability.

## 2. Materials and Experiment Methods

### 2.1. Materials

**Figure 1:**
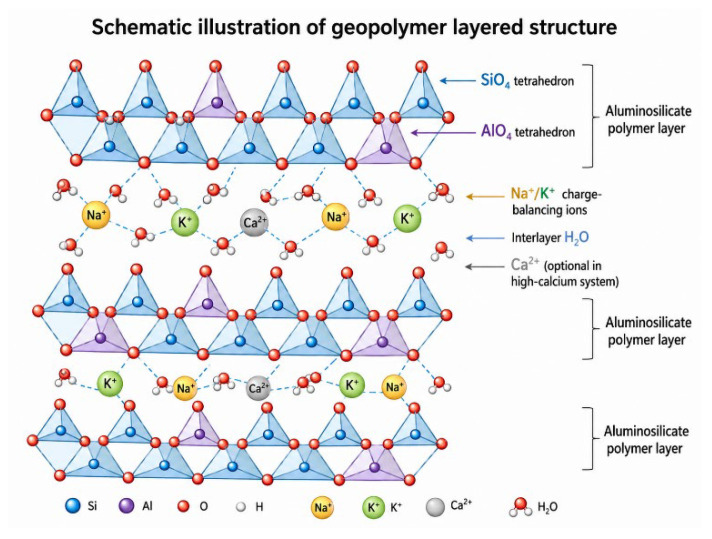
Schematic illustration of the inorganic aluminosilicate geopolymer layered structure.

The polymer component investigated in this study is the inorganic aluminosilicate geopolymer gel formed from the activation of industrial silico-aluminous wastes in Baioheng low-carbon cement. Unlike organic polymers, this geopolymer binder consists of a three-dimensional Si–O–Al network, generally expressed as Mn{–(SiO_2_)z–AlO_2_}n·wH_2_O, where M represents charge-balancing alkali ions such as Na^+^ or K^+^. In the high-calcium Baioheng cement system, Ca^2+^ also participates in the reaction, leading to the coexistence of N-A-S-H-type aluminosilicate gels and C-(A)-S-H-type calcium-bearing gels. These inorganic polymer gels constitute the main binding phase and directly affect matrix compactness, pore connectivity, and durability. To better illustrate the polymer component involved in this study, a schematic representation of the inorganic aluminosilicate geopolymer layered structure is shown in Figure 1.

**Figure 2:**
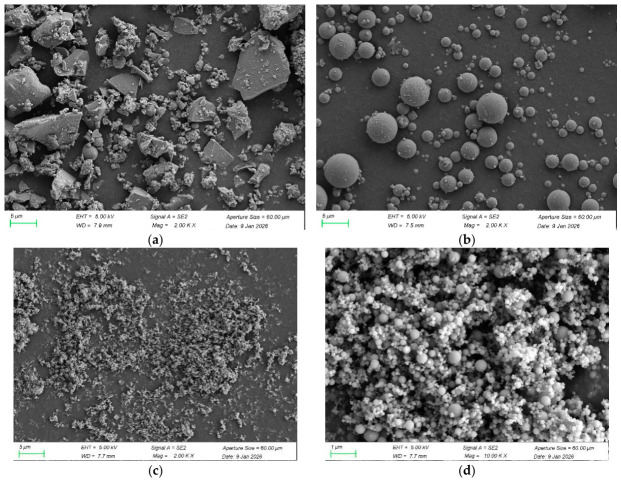
SEM images of raw materials. (**a**) B, (**b**) FA, (**c**) SF (2000×), (**d**) SF (10,000×).

**Figure 3:**
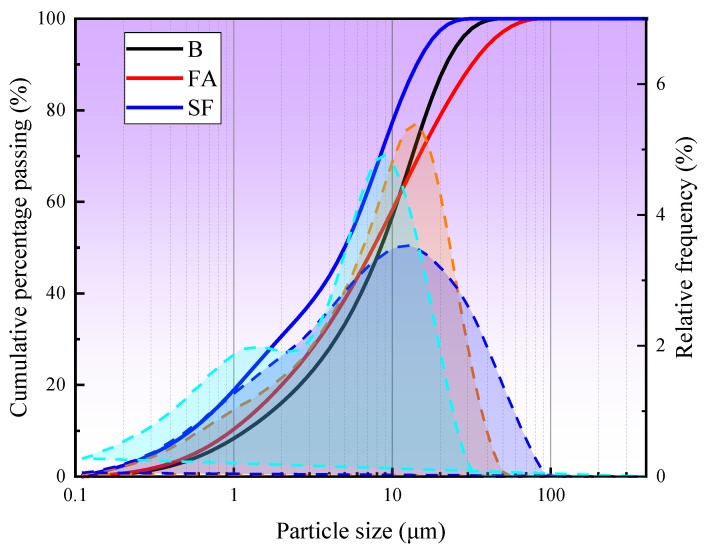
Particle size distribution diagram of raw materials.

**Figure 4:**
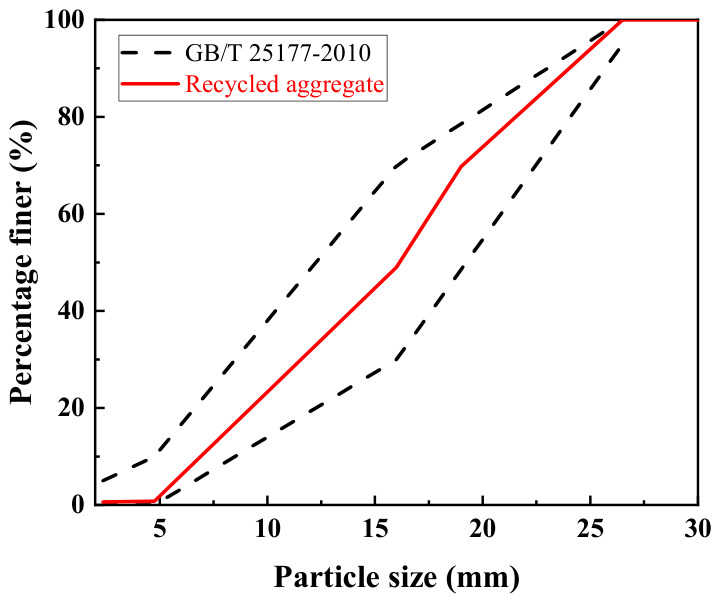
Particle size distribution curve of recycled coarse aggregate.

In this study, low-carbon cement (B) supplied by Shanghai Bai’aoheng New Materials Co., Ltd. (Shanghai, China) was used as the cementitious material. The FA used satisfied Grade I requirements, and the SF was a commercially available standard product. FA and SF were used as supplementary cementitious materials (SCMs) in this study. FA is rich in SiO_2_ and Al_2_O_3_ and can participate in secondary reactions at later ages, but its early-age reactivity is relatively low. SF contains a high amount of amorphous SiO_2_ and has a much finer particle size, which gives it stronger pozzolanic reactivity and micro-filling ability. Therefore, FA and SF were selected to regulate the inorganic aluminosilicate polymer gel structure and pore structure of Baioheng geopolymer concrete. SEM images of the three materials are shown in Figure 2, particle size distribution curves are presented in Figure 3, and their chemical compositions are listed in Table 2. The aggregates used were manufactured sand (fine aggregate) and recycled aggregate (coarse aggregate) supplied by Sichuan Xinghualutong Renewable Resources Co., Ltd. (Chengdu, China). Performance test results of the recycled coarse aggregate showed that its crushing index was 11.76%, water absorption rate was 3.9%, apparent density was 2356 kg/m^3^, and bulk density was 1578 kg/m^3^. The particle size distribution of this recycled coarse aggregate is shown in Figure 4. Bai’ao Heng cement is a prefabricated, low-carbon cementitious material prepared by alkali activation of industrial silico-aluminous wastes, with alkaline components already incorporated. In its chemical composition, SiO_2_ and Al_2_O_3_ provide the silico-aluminous source, Na_2_O and K_2_O provide the alkaline environment, and the relatively high contents of CaO and SO_3_ facilitate the formation of C-(A)-S-H gel, ettringite, and other calcium-based reaction products. Therefore, no additional NaOH or liquid silicate activators were added in this study.

### 2.2. Specimen Preparation

To investigate the effects of fly ash and silica fume contents on the mechanical properties, freeze–thaw resistance, salt-frost scaling resistance, and microstructure of low-carbon cement-based concrete, five mixture proportions were designed. The replacement ratios of fly ash to low-carbon cement were 15% and 25%, and those of silica fume to low-carbon cement were 3% and 5%. The FA replacement levels were selected as 15% and 25% to represent moderate and relatively high replacement levels, respectively. The SF replacement levels were selected as 3% and 5% to evaluate the effects of appropriate and relatively high SF contents on the gel structure and durability. During mixture design, the water-to-binder ratio, total binder content, aggregate content, and superplasticizer dosage were kept constant, so that the effects of FA and SF dosage could be compared directly. The contents of sand, recycled coarse aggregate, water-binder ratio, and superplasticizer dosage were kept constant across all mixtures. The water-binder ratio was fixed at 0.3, and the total binder content was 500 kg/m^3^. A polycarboxylate-based high-range water-reducing admixture was added at a dosage of 2.0% by mass of the total binder. The detailed mix proportion parameters are presented in Table 3. To ensure the reliability of the experimental results, three parallel specimens were used for the mechanical performance tests in each group, and the average value was taken as the test result. The freeze–thaw cycle test, one-sided salt-frost test, mass loss rate, relative dynamic elastic modulus, and chloride ion content tests were statistically analyzed based on parallel specimens or parallel samples. Error bars were added to all bar charts in the manuscript, with the error bars representing the standard deviation, to reflect the dispersion and repeatability of the experimental data.

Specimen preparation was carried out by weighing each raw material according to the mix proportions. First, the coarse and fine aggregates were mixed for 3 min to ensure uniform blending. Then, the cement was added and mixed for another 3 min. Water was added in three equal portions, with each portion mixed for 2 min, until a change in the consistency of the mixture was observed. The mixture was then poured into molds and compacted in layers by vibration. After casting, the specimens were covered with plastic film to reduce early-age moisture loss. They were demolded after being left to stand at room temperature for 24 h, and then cured in a standard curing room until the specified age. The standard curing conditions were a temperature of 20 ± 2 °C and a relative humidity of not less than 95%.

### 2.3. Experimental Methods

#### 2.3.1. Mechanical Test Method

**Figure 5:**
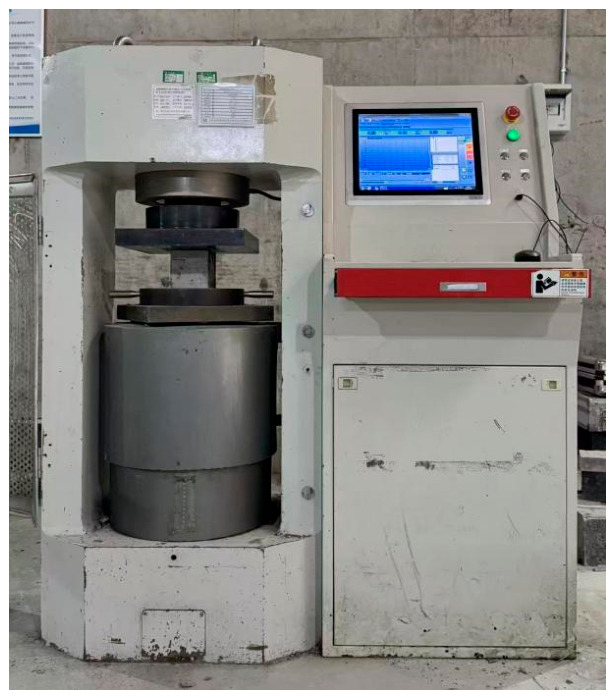
Diagram of compressive strength loading process.

The mechanical properties of the low-carbon concrete were tested in accordance with the “Standard for Test Methods of Physical and Mechanical Properties of Concrete” (GB/T 50081-2019) [30], as shown in Figure 5. The cube compressive strength test was conducted using an electro-hydraulic servo universal testing machine with a maximum testing force of 2000 kN. The specimen dimensions were 100 mm × 100 mm × 100 mm, and the loading rate was set to 0.5 MPa/s. For each macroscopic test (compressive strength, drying shrinkage, etc.), three parallel specimens were tested for each mixture, and the results were averaged. Error bars in the corresponding figures represent standard deviation. For chloride content measurements, parallel powder samples collected from the same depth were used to reduce sampling variability.

**Figure 6:**
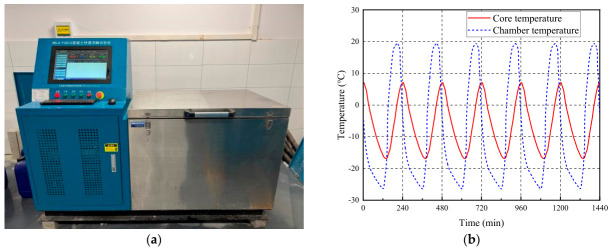
Freeze–thaw cycle test. (**a**) Freeze–thaw testing chamber, (**b**) temperature variation during freeze–thaw cycles.

Freeze–thaw cycles were set at 0, 25, 50, 75, 100, and 125 cycles in accordance with ASTM C666/C666M-15 [31], with a duration of 4.5 h per freeze–thaw cycle. The test procedure was as follows. Initial measurements at 0 cycles included mass, dynamic elastic modulus, and compressive strength. For freeze–thaw cycling, the 7 d specimens were immersed in water at 20 ± 2 °C for 4 d. The specimens subjected to 0 freeze–thaw cycles were directly measured for initial mass and dynamic elastic modulus, and the values were recorded. The remaining specimens were placed in a rapid freeze–thaw chamber, with each cycle lasting 4.5 h and the center temperature ranging from −17 °C to 5 °C. The aforementioned parameters were measured every 25 cycles. The termination conditions were completion of 125 cycles, or relative dynamic elastic modulus ≤ 60%, or mass loss rate ≥ 5%. As shown in Figure 6.

**Figure 7:**
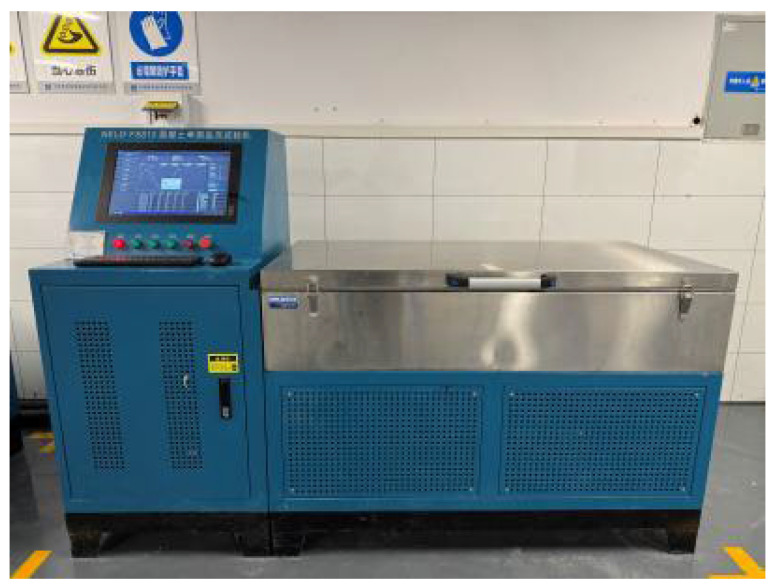
Single-sided salt freeze–thaw testing chamber.

**Figure 8:**
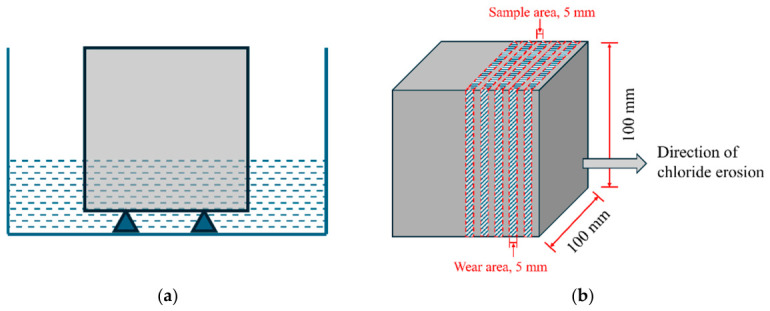
Single-sided salt freeze–thaw test. (**a**) Single-sided salt freeze–thaw cycle, (**b**) schematic diagram of concrete specimen cutting.

Referring to the standard GB/T 50082-2009 [31] “Standard Test Method for Long-Term Performance and Durability of Ordinary Concrete”, concrete specimens of size 100 mm × 100 mm × 100 mm were prepared and cured under standard conditions (20 ± 2 °C, 95% relative humidity) for 24 d. Prior to the salt-freezing (S-F) cycle test, the specimens were immersed in water for 4 d. The salt-freezing cycle test was conducted within a temperature range of –20 °C to 8 °C, as shown in Figure 7. Before the salt-freezing cycle test, five surfaces of each concrete specimen were sealed with epoxy resin to ensure unidirectional chloride ion ingress. The standard freeze–thaw test mainly simulates uniform freeze–thaw damage under water-saturated conditions, whereas the single-side salt freeze–thaw test simulates directional chloride ingress from an exposed surface. In this test, five surfaces were sealed with epoxy resin and only one surface was exposed to the salt solution. Therefore, the test better represents concrete members in cold coastal regions, tidal zones, splash zones, and deicing-salt environments, where water and chloride ions usually penetrate from the exposed surface into the interior. Salt solutions with chloride ion concentrations of 0%, 5%, 10%, 15%, and 20% were prepared as the erosion solutions. The number of salt-freezing cycles was set to 25, 50, 75, and 100. After every 25 salt-freezing cycles, the epoxy resin on the concrete surface was reapplied and the erosion solution was freshly prepared. After the salt-freezing cycle test, the epoxy resin on the concrete surface was removed to determine the compressive strength and mass loss of the concrete, and test samples were collected. Concrete samples at different erosion depths were cut from the surface subjected to chloride ion ingress using a diamond saw blade. The thickness of the worn area was 5 mm, and each concrete specimen was cut into 5 layers. The distance from the sample center to the erosion surface represents the sampling depth. The sampling depths and cutting sequence are shown in Table 4 and Figure 8, respectively. The sliced concrete samples were crushed, and the coarse aggregates were removed. Subsequently, block concrete samples and sliced mortar samples were collected. The collected block samples were crushed, ground, and sieved through a 0.3 mm aperture sieve. The resulting sliced and powder samples were used for microscopic analysis and chloride ion content testing.

#### 2.3.2. Microstructural Test Method

Scanning electron microscopy combined with energy dispersive spectroscopy (SEM-EDS, ZEISS Sigma 300, ZEISS, Oberkochen, Germany) was used for microstructural observation and elemental distribution analysis. X-ray diffraction (XRD) tests were performed using a Rigaku Ultima IV diffractometer (Rigaku, Tokyo, Japan) with a scanning rate of 5°/min over a scanning angle range of 5–90°. Computed tomography (CT) tests were conducted using a ZEISS Xradia 620 Versa high-resolution three-dimensional X-ray microscope (ZEISS, Oberkochen, Germany) for non-destructive characterization of the internal pore structure, fiber distribution, and defects within the samples. SEM-EDS, XRD, and CT were used to analyze the inorganic polymer gel structure, including elemental ratios (Si/Al, Ca/Si), the presence of C-(A)-S-H and N-A-S-H related gel products, and defect connectivity. These techniques provide indirect evidence of the polymer gel network formation. FTIR, TG-DTG, or NMR were not performed in this study, and the exact Si–O–Al bonding and gel polymerization degree will need further verification in future work.

## 3. Results and Discussion

### 3.1. Mechanical Property

#### 3.1.1. Compressive Strength

**Figure 9:**
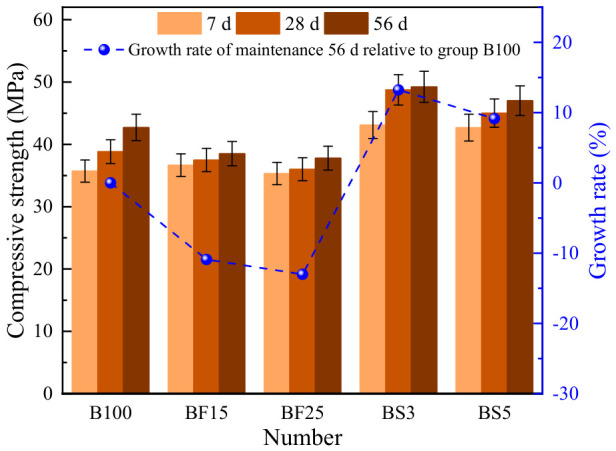
Compressive strength.

Figure 9 shows the compressive strength of geopolymer concrete with different FA and SF dosages at 56 d. It also showed the compressive strength growth rates relative to the control group B100 (42.72 MPa). The results indicated that the addition of FA significantly reduced the compressive strength of concrete. The strengths of the BF15 and BF25 groups were 38.52 MPa and 37.80 MPa, respectively. Their growth rates were −10.9% and −13.02%, respectively. Higher FA dosage led to a more significant reduction. In contrast, the addition of SF significantly increased the compressive strength. The strengths of the BS3 and BS5 groups were 49.24 MPa and 47.02 MPa, respectively. Their growth rates were +13.24% and +9.15%, respectively. The BS3 group exhibited the best improvement effect. This change was mainly attributed to the differences in pozzolanic activity and filling effect between FA and SF. FA had relatively low pozzolanic activity. Its incorporation diluted the effective concentration of cement clinker. Its dense glassy structure delayed the secondary hydration reaction. Therefore, at 56 d, the dilution effect dominated and led to a reduction in strength. From the microstructural perspective, the strength reduction in the FA-containing groups can be attributed to the combined effects of dilution and delayed reaction. The incorporation of FA reduced the amount of reactive Baioheng cement per unit volume, and the relatively low early-age reactivity of FA limited the formation of C-(A)-S-H/N-A-S-H-related gel products within the tested curing period. As observed in the SEM images, unreacted FA particles, increased pores, and a looser matrix appeared in the BF15 and BF25 groups. These features weakened the continuity of the inorganic polymer gel matrix and reduced the bonding strength between reaction products, thereby causing a decrease in compressive strength. In contrast, SF had extremely high pozzolanic activity and a large specific surface area. It could react rapidly with calcium hydroxide to generate additional C-S-H gel. It also exerted a micro-aggregate filling effect to optimize the interfacial transition zone. As a result, SF significantly improved the compactness and compressive strength of concrete. The slightly lower strengthening effect of the BS5 group compared with the BS3 group may be associated with local structural defects observed in the microstructural analysis. However, this explanation requires further verification through fresh property or rheological tests.

#### 3.1.2. Freeze–Thaw Cycle Test

**Figure 10:**
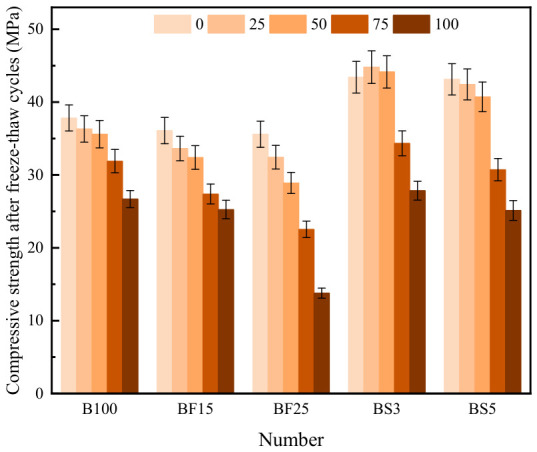
Changes in compressive strength before and after freeze–thaw cycles.

Figure 10 shows the compressive strength evolution of geopolymer concrete with different FA and SF dosages. The concrete was subjected to 0, 25, 50, 75, and 100 freeze–thaw cycles. As the number of freeze–thaw cycles increased, the compressive strength of all concrete groups showed a gradual decreasing trend. However, the response to freeze–thaw damage varied significantly depending on the type and dosage of the admixture. For the FA groups, the frost resistance of the concrete deteriorated continuously as the FA content increased. When the number of freeze–thaw cycles reached 100, the compressive strength of the control group B100 was 26.69 MPa. The compressive strengths of the BF15 and BF25 groups decreased to 25.26 MPa and 13.79 MPa, respectively. Among these, the BF25 group exhibited the most severe strength loss. Its strength decreased by 48.3% compared with the control group. This severe strength loss was further supported by the SEM and CT observations. The SEM images of BF25 showed a loose matrix, unreacted FA particles, and weak connections between reaction products, while the CT results indicated the formation of local defects and connected pore networks. During freeze–thaw cycling, water in these connected pores repeatedly froze and expanded, generating internal frost-heave pressure. The pre-existing pores and weak interfaces therefore became preferential sites for crack initiation and propagation. As a result, microcracks gradually connected with each other, leading to serious internal damage and a marked reduction in compressive strength after 100 cycles. This phenomenon was mainly attributed to the low pozzolanic activity of FA. Its secondary hydration reaction proceeded slowly. This resulted in a higher internal porosity and a coarser pore structure in the concrete. Under repeated freeze–thaw cycles, microcracks were more likely to initiate and propagate. This ultimately led to a significant deterioration in strength. For the SF groups, the change in compressive strength followed a non-linear trend. It first increased and then decreased. After 100 freeze–thaw cycles, the compressive strength of the BS3 group was 27.85 MPa. This was slightly higher than that of the control group B100. The BS3 group therefore demonstrated good freeze–thaw resistance. In contrast, the compressive strength of the BS5 group was 25.13 MPa, which was lower than that of the control group. When the SF content was 3%, its high pozzolanic activity and micro-aggregate filling effect effectively optimized the pore structure. They also improved the compactness of the interfacial transition zone. As a result, the ability of the concrete to resist freeze–thaw damage was enhanced. However, when the SF content was increased to 5%, the improvement in freeze–thaw resistance became weaker. This may be related to the local defects and increased pores observed in the SEM and CT results. However, since fresh properties were not measured in this study, the possible effects of dispersion and workability should be regarded as assumptions that require further verification. This weakened the positive effect of SF on freeze–thaw resistance. In summary, an increase in FA content continuously aggravated freeze–thaw damage. In contrast, SF could effectively mitigate the deterioration of compressive strength caused by freeze–thaw cycles only at an appropriate dosage. This result provided an important reference for the durability design of geopolymer concrete in cold environments.

**Figure 11:**
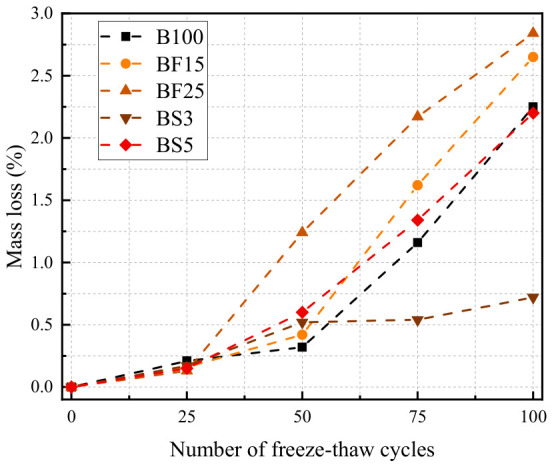
Changes in mass of geopolymer concrete before and after freeze–thaw cycles.

Figure 11 shows the mass loss evolution of geopolymer concrete with different FA and SF dosages. The concrete was subjected to 0, 25, 50, 75, and 100 freeze–thaw cycles. Overall, as the number of freeze–thaw cycles increased, the mass loss of all concrete groups showed a gradual increasing trend. However, the resistance to freeze–thaw scaling varied significantly depending on the type and dosage of the admixture. For the groups with varying FA content, the differences in mass loss among all concrete groups were small at the initial stage of freeze–thaw cycles. However, when the number of cycles increased to more than 50, the BF25 group, which had a higher FA content, showed an accelerated increasing trend. When the number of freeze–thaw cycles reached 100, the mass loss of the control group B100 was 2.25%. The mass loss of the BF15 group was 2.65%. The mass loss of the BF25 group was 2.84%. These results indicated that a higher FA content led to more severe surface scaling of the concrete. At 75 freeze–thaw cycles, the mass losses of the B100, BF15, and BF25 groups were 1.16%, 1.62%, and 2.17%, respectively. These values also showed an increasing trend with higher FA content. This phenomenon was consistent with the compressive strength results described above. It further confirmed that a high FA content led to a coarser pore structure and lower compactness in the concrete. As a result, surface scaling was more likely to occur under repeated freeze–thaw cycles. For the SF groups, the change in mass loss showed a distinct non-linear characteristic. Among all groups, the BS3 group exhibited the best resistance to freeze–thaw scaling. After 25, 50, 75, and 100 freeze–thaw cycles, its mass losses were 0.17%, 0.52%, 0.54%, and 0.72%, respectively. These values were much lower than those of the control group B100 at the corresponding cycle numbers. Notably, from 50 to 75 cycles, the mass loss of the BS3 group increased only from 0.52% to 0.54%. It almost did not change. This demonstrated its excellent freeze–thaw stability. For the BS5 group, the mass losses after 25, 50, 75, and 100 freeze–thaw cycles were 0.15%, 0.60%, 1.34%, and 2.20%, respectively. From 75 to 100 cycles, the rate of mass loss accelerated significantly. The final mass loss of the BS5 group was close to that of the B100 group (2.25%). However, it was significantly higher than that of the BS3 group (0.72%). These results indicated that a 3% SF content significantly optimized the pore structure of the concrete. It also reduced pore connectivity. As a result, surface scaling caused by freeze–thaw cycles was effectively inhibited. When the SF content was increased to 5%, the improvement effect weakened. This was likely due to uneven dispersion or increased water demand. Consequently, scaling intensified in the later stage of freeze–thaw cycles. Considering both mass loss and compressive strength, the addition of FA generally deteriorated the freeze–thaw resistance of the concrete. A higher FA content led to more severe deterioration. In contrast, a 3% SF content significantly enhanced the ability of the concrete to resist freeze–thaw scaling and strength deterioration. Therefore, incorporating an appropriate amount of SF (3%) was an effective way to improve the freeze–thaw resistance of geopolymer concrete.

**Figure 12:**
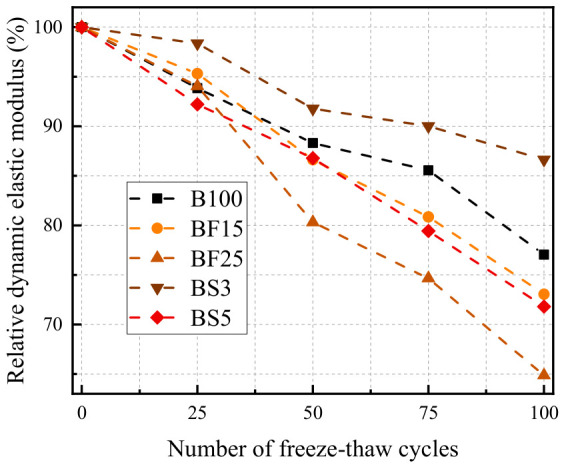
Changes in relative dynamic elastic modulus of geopolymer concrete.

Figure 12 shows the evolution of the relative dynamic elastic modulus of geopolymer concrete with different FA and SF dosages. The concrete was subjected to 0, 25, 50, 75, and 100 freeze–thaw cycles. As the number of freeze–thaw cycles increased, the relative dynamic elastic modulus of all concrete groups showed a gradual decreasing trend. However, the resistance to internal damage varied significantly depending on the type and dosage of the admixture. For the FA groups, when the number of freeze–thaw cycles reached 100, the relative dynamic elastic modulus of the control group B100 was 77.048%. The values for the BF15 and BF25 groups decreased to 73.056% and 64.860%, respectively. At 50 and 75 cycles, a similar decreasing trend was observed with increasing FA content. This indicated that a high FA content led to a coarser pore structure and a weaker interfacial transition zone in the concrete. As a result, microcracks were more likely to initiate and propagate. For the SF groups, the BS3 group exhibited the best resistance to internal freeze–thaw damage. After 25, 50, 75, and 100 cycles, its relative dynamic elastic moduli were 98.360%, 91.780%, 90.016%, and 86.640%, respectively. At each stage, these values were significantly higher than those of the control group B100. Notably, from 50 to 75 cycles, the reduction was only 1.764 percentage points. This demonstrated excellent internal structural stability of the BS3 group. In contrast, the values for the BS5 group after the same number of cycles were 92.208%, 86.780%, 79.428%, and 71.808%, respectively. Its final value was close to that of the B100 group but was significantly lower than that of the BS3 group. Considering the three indicators (relative dynamic elastic modulus, compressive strength, and mass loss), an increase in FA content continuously deteriorated the freeze–thaw resistance of the concrete. In contrast, a 3% SF content significantly enhanced the ability of the concrete to resist internal freeze–thaw damage and surface scaling. This improvement was attributed to the pore filling and interfacial transition zone optimization effects provided by the high pozzolanic activity of SF. However, when the SF content was increased to 5%, the improvement effect weakened. This weakening was possibly related to a decrease in dispersion uniformity and an increase in water demand.

#### 3.1.3. Single-Side Salt Freeze–Thaw Cycle Test

**Figure 13:**
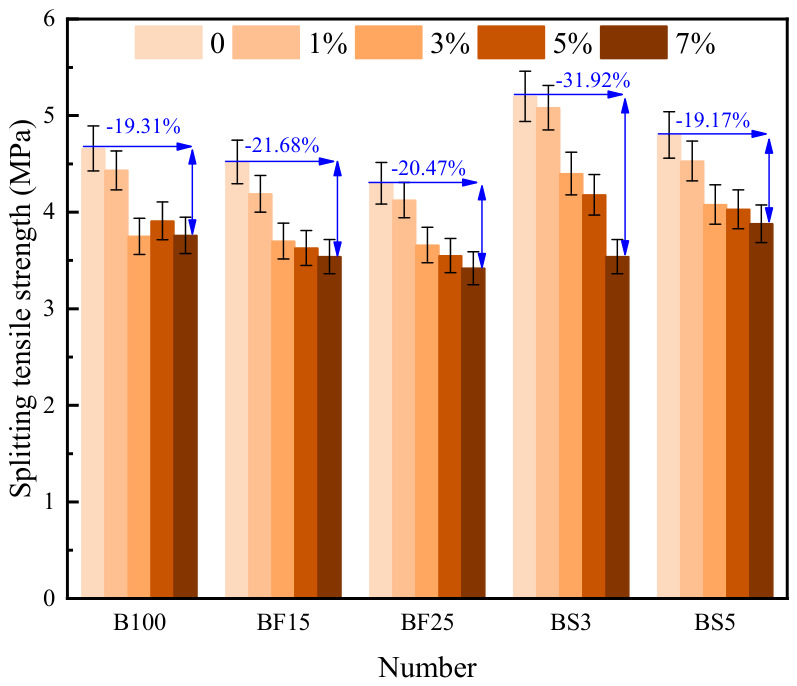
Mass loss of concrete under single-sided salt freeze–thaw cycles at different salt concentrations.

Compared with the standard freeze–thaw test, the single-side salt freeze–thaw test combines directional chloride ingress, surface scaling, and freeze–thaw damage, which is closer to the exposure condition of concrete structures in cold coastal environments. Figure 13 shows the variation in splitting tensile strength of geopolymer concrete with different FA and SF dosages. The concrete was tested under a single-sided salt freeze–thaw cycle. The chloride ion concentration of the salt solution increased from 0% to 7%. Overall, as the salt ion concentration increased, the splitting tensile strength of all concrete groups showed a gradual decreasing trend. However, the resistance to salt-frost erosion varied significantly depending on the type and dosage of the admixture. When the salt ion concentration was 0%, the splitting tensile strengths of the B100, BF15, BF25, BS3, and BS5 groups were 4.66, 4.52, 4.30, 5.20, and 4.80 MPa, respectively. When the salt ion concentration increased to 7%, the splitting tensile strengths of all groups decreased. The values for the B100, BF15, BF25, BS3, and BS5 groups were 3.76, 3.54, 3.42, 3.54, and 3.88 MPa, respectively. Compared with their respective strengths at 0% salt ion concentration, the strength reduction for the B100 group was 19.31%. The reduction for the BF15 group was 21.68%. The reduction for the BF25 group was 20.47%. The reduction for the BS3 group was 31.92%. The reduction for the BS5 group was 19.17%. It could be observed that the BS3 group exhibited the largest relative strength reduction of 31.92%. However, its initial splitting tensile strength at 0% salt concentration was the highest among all mixtures, reaching 5.20 MPa. Therefore, even after salt-freezing erosion, its absolute strength remained at 3.54 MPa, which was still comparable to or higher than several other groups. This indicates that the dense inorganic gel matrix formed with 3% SF provided a high initial load-bearing capacity. However, under single-side salt freeze–thaw conditions, the denser structure may also restrict the release of pore solution and lead to higher local pore pressure, resulting in a larger relative strength loss. In contrast, the BF25 group had the lowest absolute strength at 7% salt ion concentration, which was only 3.42 MPa. The decrease in splitting tensile strength with increasing salt ion concentration was attributed to several main reasons. Compared with pure water, chloride solution changes the freezing behavior of the pore solution. The presence of chloride ions lowers the freezing point and allows part of the pore solution to remain unfrozen at lower temperatures. During freezing, ice formation increases the ion concentration in the remaining liquid phase, producing osmotic pressure and concentration gradients. In addition, ice expansion and possible salt crystallization jointly increase the pressure acting on pore walls. When the pore network is connected or microcracks already exist, these pressures can promote crack propagation and accelerate freeze–thaw damage. Therefore, chloride ions affect not only chemical binding and transport behavior, but also the pore pressure development during salt freeze–thaw cycles. These pressures exacerbated microcrack propagation. In the high-calcium alkali-activated cementitious system used in this study, chloride salt erosion may reduce material performance mainly by changing the pore solution characteristics, accelerating freeze–thaw damage accumulation, and affecting the stability of reaction products and pore structure. Meanwhile, chloride ions may be adsorbed by C-(A)-S-H/N-A-S-H gels or partially bound by Al-bearing reaction products. However, the specific chloride binding forms in this system require further verification by additional tests. As the salt ion concentration increased, these destructive effects intensified. This led to an increase in porosity and a decrease in the bond strength between the aggregate and the paste. Consequently, the strength continued to decrease. Notably, the BS3 group had the highest initial strength but also the largest reduction. This might have been because SF increased the compactness of the concrete. However, in the single-sided salt freeze–thaw cycle environment, the dense structure prevented the effective release of pore water. As a result, it endured higher frost heave stresses. In summary, an increase in salt ion concentration significantly deteriorated the splitting tensile strength of geopolymer concrete.

**Figure 14:**
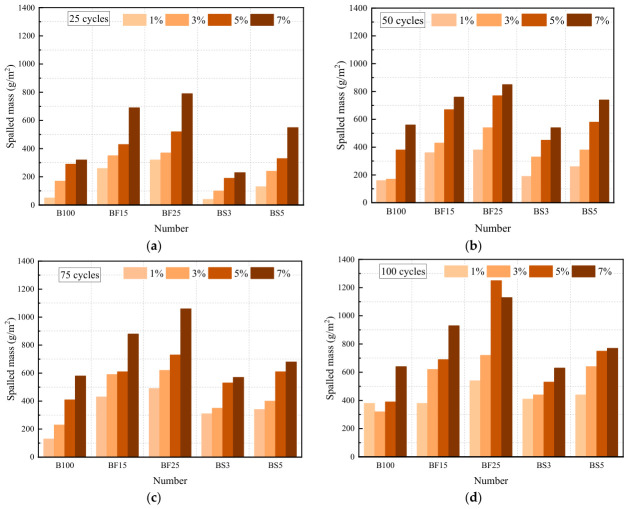
Single-side salt mass loss of concrete at various salt concentrations. (**a**) 25 cycles, (**b**) 50 cycles, (**c**) 75 cycles, (**d**) 100 cycles.

The changes in surface scaling mass of geopolymer concrete with different FA and SF dosages are shown in Figure 14. The concrete was tested under single-sided salt freeze–thaw cycles. The number of freeze–thaw cycles and the chloride ion concentration of the salt solution increased. As the number of freeze–thaw cycles and the salt ion concentration increased, the scaling mass of all concrete groups showed a gradual increasing trend. However, the resistance to salt-frost scaling varied significantly depending on the type and dosage of the admixture. For the FA groups, at the same number of freeze–thaw cycles, the scaling mass increased significantly with higher salt ion concentration. Taking the BF25 group as an example, at 25 freeze–thaw cycles, the scaling masses under salt ion concentrations of 1%, 3%, 5%, and 7% were 320, 370, 790, and 770 g/m^2^, respectively. When the number of freeze–thaw cycles increased to 100, the corresponding scaling masses increased to 540, 720, 1250, and 1130 g/m^2^, respectively. At 100 freeze–thaw cycles and a salt ion concentration of 7%, the scaling mass of the control group B100 was 640 g/m^2^. Under the same conditions, the scaling masses of the BF15 and BF25 groups were 930 and 1130 g/m^2^, respectively. This indicated that a higher FA content led to more severe salt-frost scaling.

In terms of resistance to salt-frost scaling, the SF groups performed significantly better than the FA groups. Among them, the BS3 group exhibited the best scaling resistance. Under salt ion concentrations of 1%, 3%, 5%, and 7%, as the number of freeze–thaw cycles increased from 25 to 100, the scaling masses of the BS3 group were 410, 440, 530, and 630 g/m^2^, respectively. These values were consistently significantly lower than those of the BS5 group, which were 440, 640, 750, and 770 g/m^2^, respectively. This indicated that when the SF content was increased to 5%, its improvement effect weakened. In summary, the addition of FA significantly deteriorated the scaling resistance of concrete under salt-frost environments. A higher FA content led to greater scaling mass and more severe salt-frost damage. In contrast, the addition of SF effectively enhanced the salt-frost scaling resistance of concrete. Among all groups, the BS3 group with 3% SF showed the best performance. It maintained low scaling mass under all salt ion concentrations and freeze–thaw cycle numbers. However, when the SF content was increased to 5%, the improvement effect weakened. Its scaling mass was significantly higher than that of the BS3 group. Under some conditions, it even approached or exceeded that of the control group B100. These results indicated that incorporating an appropriate amount of SF into geopolymer concrete was an effective way to improve salt-frost resistance. However, the SF content needed to be controlled within a reasonable range. The application of FA, on the other hand, required careful evaluation of its suitability in salt-frost environments.

**Figure 15:**
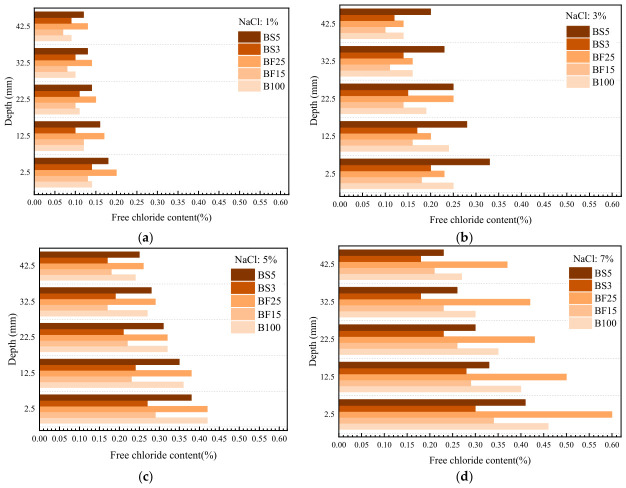
Effect of the number of single-side salt freeze–thaw cycles on Cl^-^ transport in concrete. (**a**) 1% NaCl solution, (**b**) 3% NaCl solution, (**c**) 5% NaCl solution, (**d**) 7% NaCl solution.

Figure 15a–d showed the distribution of chloride ion content in concrete at different sampling depths. The concrete was tested under single-sided salt freeze–thaw cycles. The number of freeze–thaw cycles was 100. Different salt solution concentrations (1%, 3%, 5%, and 7%) were applied. The sampling depths were 2.5 mm, 12.5 mm, 22.5 mm, 32.5 mm, and 42.5 mm. Overall, as the sampling depth increased, the chloride ion content of all concrete groups showed a gradual decreasing trend. This indicated that chloride ion transport in concrete followed a diffusion law from the surface to the interior. Meanwhile, as the salt solution concentration increased, the chloride ion content at the same depth also increased accordingly. When the salt solution concentration was 1%, the chloride ion contents at the surface layer (2.5 mm) for the B100, BF15, BF25, BS3, and BS5 groups were 0.14%, 0.13%, 0.20%, 0.14%, and 0.18%, respectively. As the depth increased to 42.5 mm, these values decreased to 0.09%, 0.07%, 0.13%, 0.09%, and 0.12%, respectively. Among all groups, the BF25 group showed higher chloride ion content at all depths than the other groups. This indicated that a high FA content increased the chloride ion penetration depth. When the salt solution concentration was 3%, the chloride ion content of all groups increased significantly compared with the 1% condition. At the surface layer (2.5 mm), the chloride ion contents of the B100, BF15, BF25, BS3, and BS5 groups were 0.25%, 0.18%, 0.23%, 0.20%, and 0.33%, respectively. Among these, the BS5 group had the highest surface content. Notably, the BF25 group showed an abnormally high value of 0.25% at a depth of 22.5 mm. This value was higher than its surface content. This abnormal chloride distribution may be related to the increased pore connectivity and freeze–thaw-induced microcracks caused by the high FA content in the BF25 group. These defects may provide preferential pathways for chloride solution to migrate into the interior. In addition, surface scaling during salt freeze–thaw cycles may remove part of the chloride-enriched surface layer, resulting in a relatively lower chloride content near the exposed surface. Therefore, this abnormal point was regarded as a local fluctuation rather than the general chloride transport trend. When the salt solution concentration was 5%, the chloride ion content increased further. At the surface layer (2.5 mm), the chloride ion contents of the B100, BF15, BF25, BS3, and BS5 groups were 0.42%, 0.29%, 0.42%, 0.27%, and 0.38%, respectively. The B100 and BF25 groups had the same surface content. However, the BF25 group showed higher chloride ion content than the B100 group at depths ranging from 12.5 mm to 42.5 mm. This indicated that an increase in FA content promoted chloride ion penetration. When the salt solution concentration was 7%, the chloride ion content reached its highest level. At the surface layer (2.5 mm), the chloride ion contents of the B100, BF15, BF25, BS3, and BS5 groups were 0.46%, 0.34%, 0.60%, 0.41%, and 0.36%, respectively. Among these, the BF25 group had the highest surface content, reaching 0.60%. Its chloride ion content was also generally higher than that of the other groups at depths ranging from 12.5 mm to 42.5 mm. This further confirmed that a high FA content significantly reduced the ability of concrete to resist chloride ion erosion. In contrast, the BS3 group showed relatively low chloride ion content under all salt concentrations. This indicated that an appropriate amount of SF could increase the compactness of concrete. It also enhanced the resistance to chloride ion penetration. Overall, the chloride ion content decreased with increasing depth. It increased with increasing salt concentration. An increase in FA content aggravated chloride ion penetration. In contrast, the incorporation of 3% SF effectively inhibited chloride ion ingress and improved the erosion resistance. Moreover, for the high-calcium alkali-activated cementitious system used in this study, chloride binding and transport are mainly governed by the combined effects of reaction product composition and pore structure. C-(A)-S-H/N-A-S-H gels can provide adsorption sites for chloride ions, while Al-bearing reaction products may contribute to chloride binding. In addition, the changes in pore structure induced by FA and SF further affect the migration pathways and penetration depth of chloride ions.

### 3.2. Microstructural Analysis

#### 3.2.1. SEM Analysis

**Figure 16:**
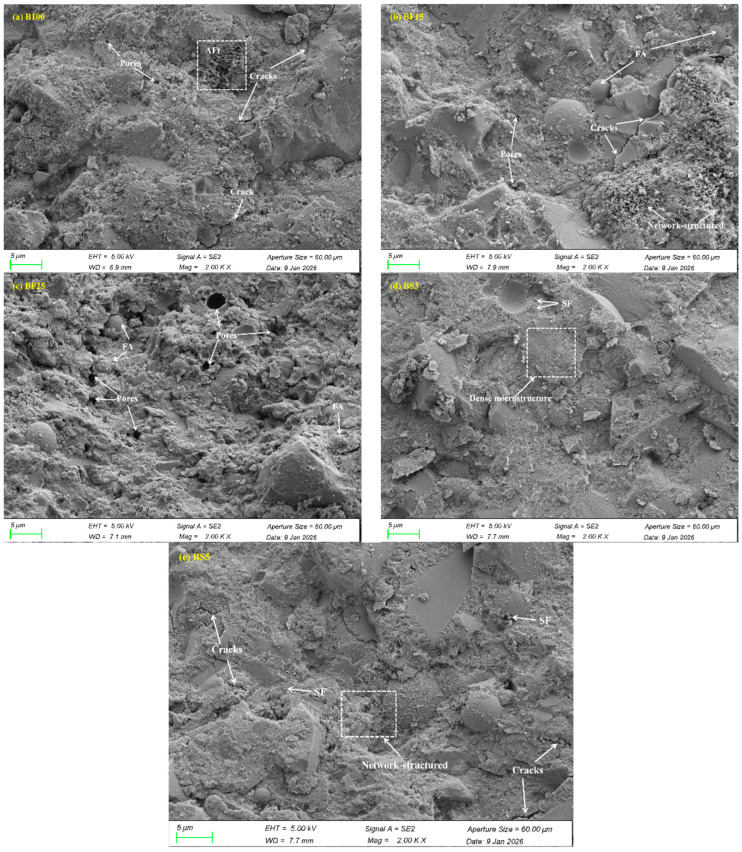
SEM image of geopolymer concrete.

As shown in Figure 16, the control group B100 had a relatively dense microstructure. The hydration products were uniformly distributed. No obvious macroscopic cracks or pores were observed. With an increase in FA content, the microstructures of the BF15 and BF25 groups gradually became looser. In the BF15 group, a small number of unreacted FA particles appeared. The compactness of the matrix decreased. The BF25 group showed more significant structural deterioration. A large number of pores and unhydrated FA particles were visible in the matrix. The connections between hydration products were weak. This explained the reduction in compressive strength and freeze–thaw resistance of this group. For the SF groups, the BS3 group exhibited a relatively dense microstructure. The hydration products were tightly bonded. The porosity was significantly reduced. This indicated that a 3% SF content could effectively fill pores and optimize the matrix structure. In contrast, obvious cracks and network-like structures were observed in the images of the BS5 group. SF agglomerates were also present. This indicated that when the SF content was increased to 5%, local microcracks and heterogeneous structures appeared in the matrix. These defects may weaken the beneficial filling effect of SF. However, whether they were caused by SF agglomeration or changes in fresh properties requires further verification. As a result, it had a negative impact on the microstructure. This was consistent with the performance fluctuations observed in the mechanical properties and durability tests of this group. In summary, an appropriate amount of FA and SF could improve the microstructure of geopolymer concrete to some extent. However, excessively high dosages led to a loose structure, increased porosity, and even microcracks. This, in turn, deteriorated the macroscopic performance of the material.

**Figure 17:**
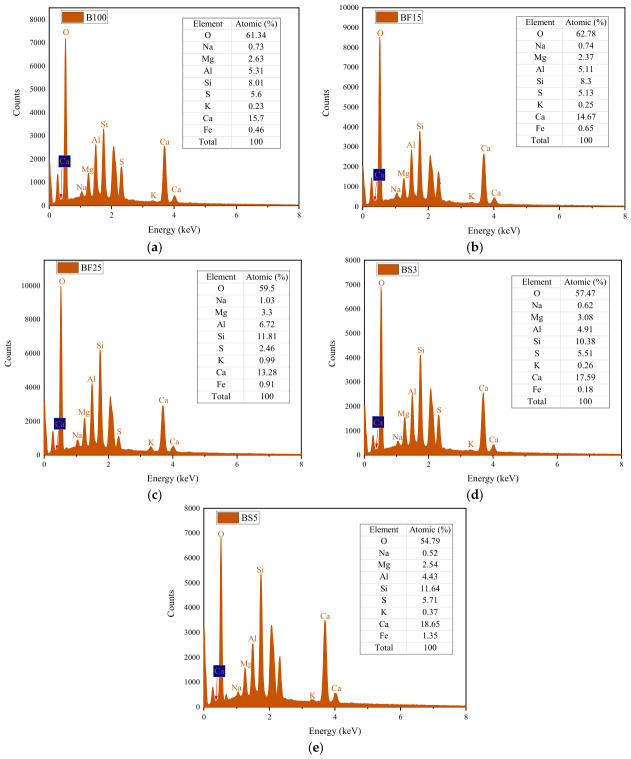
EDS image of geopolymer concrete. (**a**) B100, (**b**) BF15, (**c**) BF25, (**d**) BS3, (**e**) BS5.

**Figure 18:**
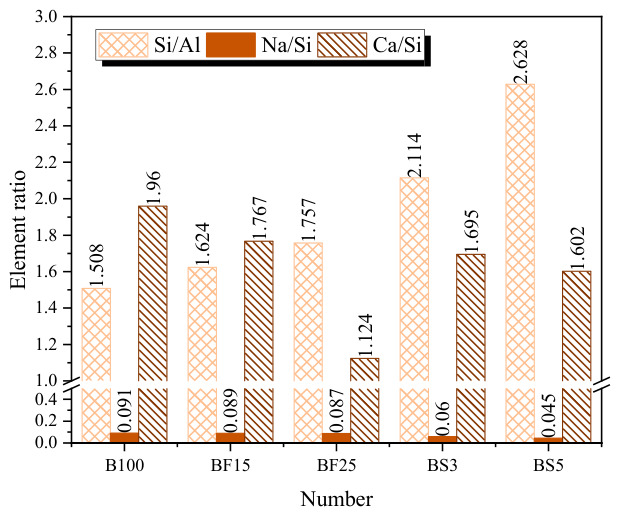
Element ratio of geopolymer concrete.

As shown in Figure 17 and Figure 18, with an increase in FA content, the Si/Al ratio gradually increased. It rose from 1.508 in the B100 group to 1.624 in the BF15 group and 1.757 in the BF25 group. The Ca/Si ratio decreased significantly from 1.960 to 1.767 and 1.124. The Na/Si ratio decreased slightly from 0.091 to 0.089 and 0.087. These changes were attributed to the fact that FA was rich in reactive SiO_2_ and Al_2_O_3_. Its introduction increased the relative contents of silicon and aluminum in the system. Meanwhile, the secondary hydration reaction of FA consumed the calcium hydroxide generated by cement hydration. This led to a relative reduction in calcium. Therefore, the Si/Al ratio increased and the Ca/Si ratio decreased. In addition, the dilution effect of FA also reduced the proportion of cement clinker per unit volume. This further intensified the decrease in the Ca/Si ratio. For the SF groups, as the SF content increased from 3% to 5%, the Si/Al ratio rose from 2.114 to 2.628. The Ca/Si ratio decreased slightly from 1.695 to 1.602. Compared with the control group B100, whose Si/Al and Ca/Si ratios were 1.508 and 1.960, respectively, the BS3 group showed a higher Si/Al ratio of 2.114 and a lower Ca/Si ratio of 1.695. The increase in Si/Al ratio indicates that the reactive SiO_2_ introduced by SF promoted the formation of Si-rich aluminosilicate gel products. Meanwhile, the decrease in the Ca/Si ratio suggests that part of the calcium was consumed to form C-(A)-S-H-type gels with relatively lower Ca/Si ratios. This balanced gel composition may improve the continuity and compactness of the inorganic polymer matrix, which explains the improved compressive strength and freeze–thaw resistance of the BS3 group. The Na/Si ratio decreased from 0.060 to 0.045. The extremely high amorphous SiO_2_ content in SF significantly increased the silicon content of the system. This caused a substantial increase in the Si/Al ratio. At the same time, the high pozzolanic activity of SF enabled it to react rapidly with calcium hydroxide to form additional C-S-H gel. This reduced the Ca/Si ratio. The continuous decrease in the Na/Si ratio may have been related to the dilution of the sodium ion concentration in the system by SF. It may also have been related to the promotion of sodium participation in the formation of a more stable gel structure. Notably, the Si/Al ratios of the BS3 and BS5 groups (2.114 and 2.628) were significantly higher than that of the BF25 group (1.757). Their Ca/Si ratios (1.695 and 1.602) were also higher than that of the BF25 group (1.124). This indicated that SF had a stronger regulatory effect on increasing the Si/Al ratio than FA. However, its degree of calcium consumption was relatively weaker. This may have been because SF did not introduce aluminum elements. Its high reactivity was mainly directed at calcium hydroxide. The changes in the elemental ratios shown in Figure 18 were consistent with the microstructural morphology in Figure 16 and the macroscopic mechanical properties. A higher Si/Al ratio and a moderate Ca/Si ratio favored the formation of a dense gel structure. This, in turn, enhanced the strength and durability of the concrete. Overall, FA and SF affected the XRD peak evolution in different ways. The incorporation of FA reduced the peak intensities of ettringite and C-(A)-S-H-related reaction products because of the dilution effect and the slow early reaction of FA. The calcite peak also weakened, which may be related to the reduction in available calcium sources for carbonation. In contrast, SF promoted the formation of low-crystalline C-(A)-S-H-type gel products due to its high amorphous SiO_2_ content and pozzolanic reactivity. Therefore, the C-(A)-S-H-related broad peak became more pronounced in the SF groups. The ettringite peak was also enhanced, indicating that SF promoted early reaction and sulfate-related product formation. These XRD changes are consistent with the SEM-EDS results and explain the improved matrix compactness of the BS3 group. The C-S-H, ettringite, anhydrite, and calcite phases observed in XRD do not indicate a conventional OPC system, but reflect the combined effects of silico-aluminate polymerization and calcium-based reactions in high-calcium alkali-activated industrial waste cementitious materials.

#### 3.2.2. XRD Analysis

**Figure 19:**
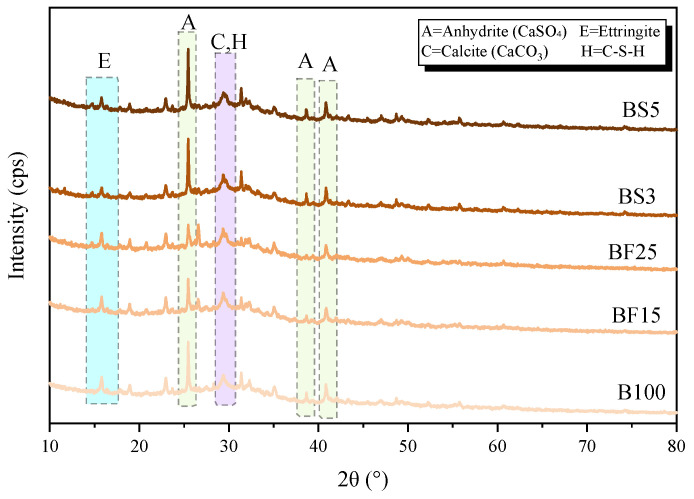
XRD of geopolymer concrete.

As shown in Figure 19, the main hydration products of geopolymer concrete included anhydrite, calcite, ettringite, and C-S-H gel. As the FA content increased from 15% to 25%, the characteristic peak intensities of ettringite and C-S-H gel gradually decreased. This indicated that the addition of FA diluted the effective concentration of cement clinker. The early pozzolanic activity of FA was low. Its secondary hydration reaction proceeded slowly. These factors led to a reduction in the formation of ettringite and C-S-H gel. Meanwhile, the diffraction peak intensity of anhydrite decreased slightly. This may have been related to the dilution or adsorption of sulfate ions in the system by FA. The characteristic peak intensity of calcite weakened with increasing FA content. This was because FA reduced the proportion of cement clinker per unit volume. This, in turn, reduced the content of calcium hydroxide available for carbonation. Ultimately, this led to a decrease in the formation of calcite. As the SF content increased from 3% to 5%, the characteristic peak intensity of C-S-H gel increased significantly. The diffuse peak shape became broader. This was attributed to the high pozzolanic activity of SF. Its reactive SiO_2_ reacted rapidly with the calcium hydroxide generated by cement hydration. This produced a large amount of low-crystallinity or amorphous C-S-H gel. The characteristic peak intensity of ettringite also increased after the incorporation of SF. This may have been related to the promotion of early hydration and the accelerated formation of ettringite by SF. The diffraction peak intensity of anhydrite decreased with increasing SF content. This indicated that the addition of SF promoted the dissolution of anhydrite and its participation in the hydration reaction. The characteristic peak intensity of calcite in the SF groups was significantly lower than that in the control group. This was because SF consumed the calcium hydroxide in the system. This reduced the opportunity for carbonation to form calcite. Notably, the C-S-H gel peak intensity in the BS5 group was higher than that in the BS3 group. However, the SEM results showed the presence of SF agglomerates and microcracks in the BS5 group. This indicated that although excessive SF promoted the formation of C-S-H, uneven dispersion introduced structural defects. This, in turn, was detrimental to the macroscopic performance. The above XRD phase evolution patterns were consistent with the EDS elemental ratios and SEM microstructural observations. Together, they revealed the regulatory mechanisms of FA and SF on the hydration product composition of geopolymer concrete. The C-S-H, ettringite, anhydrite, and calcite phases observed in XRD do not indicate a conventional OPC system, but reflect the combined effects of silico-aluminate polymerization and calcium-based reactions in high-calcium alkali-activated industrial waste cementitious materials.

#### 3.2.3. CT Analysis

**Figure 20:**
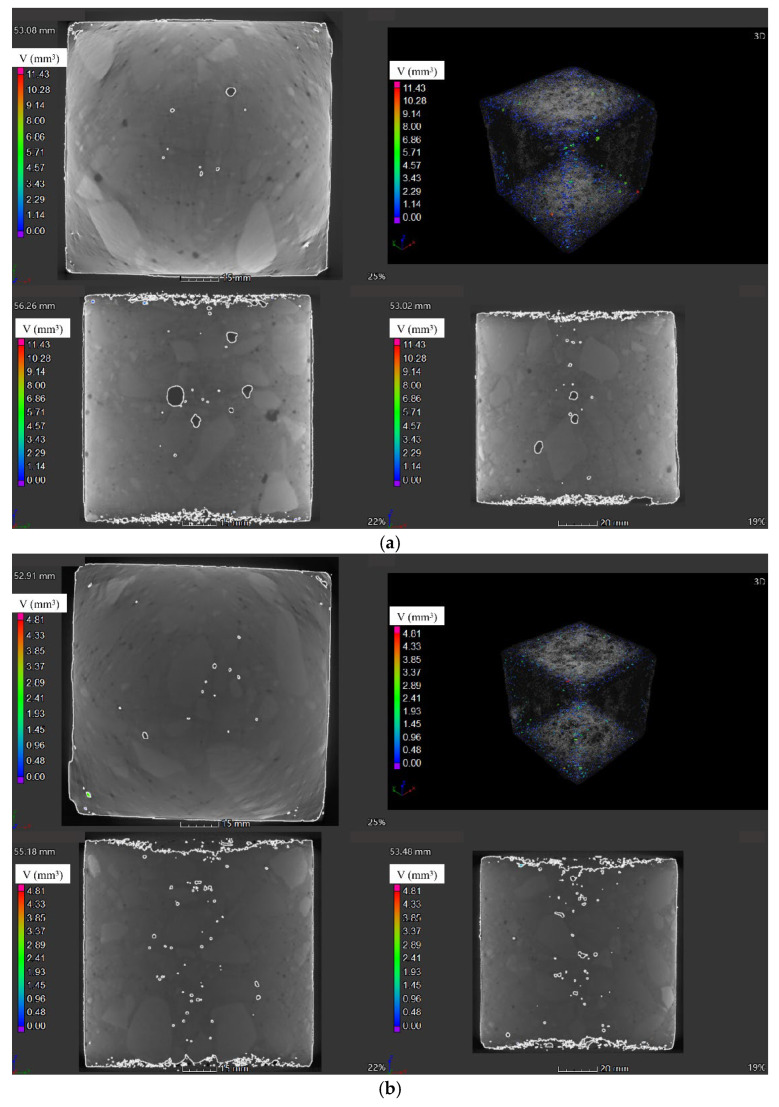

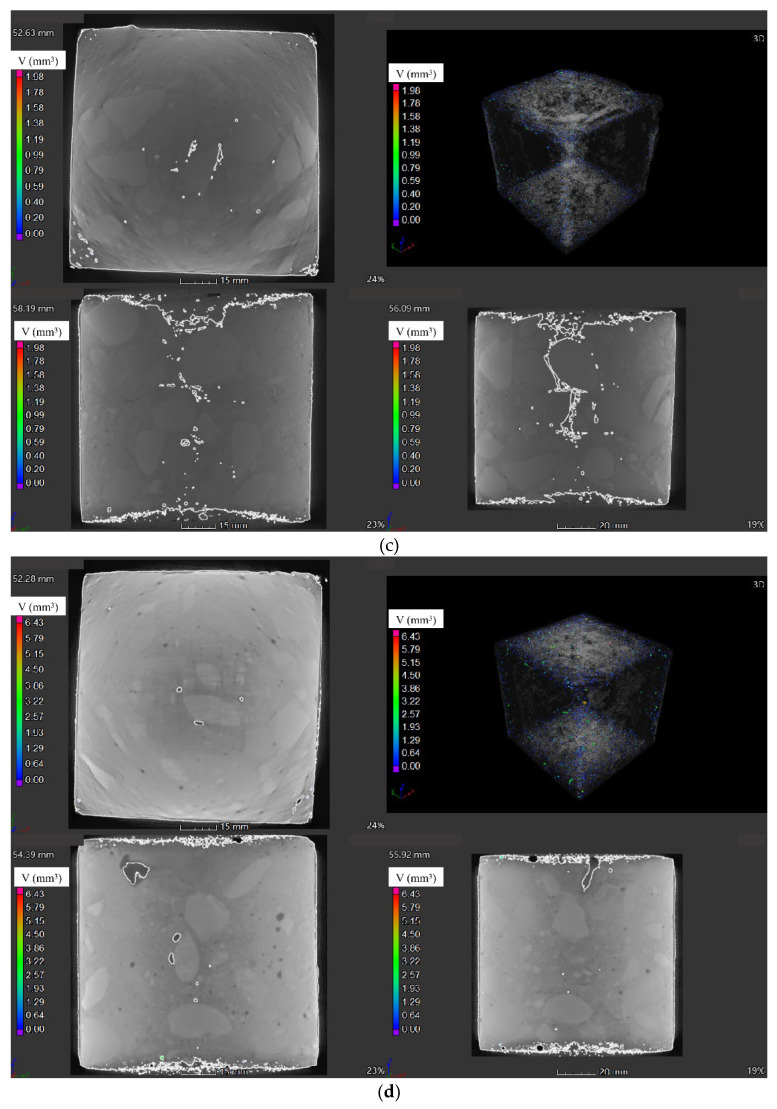

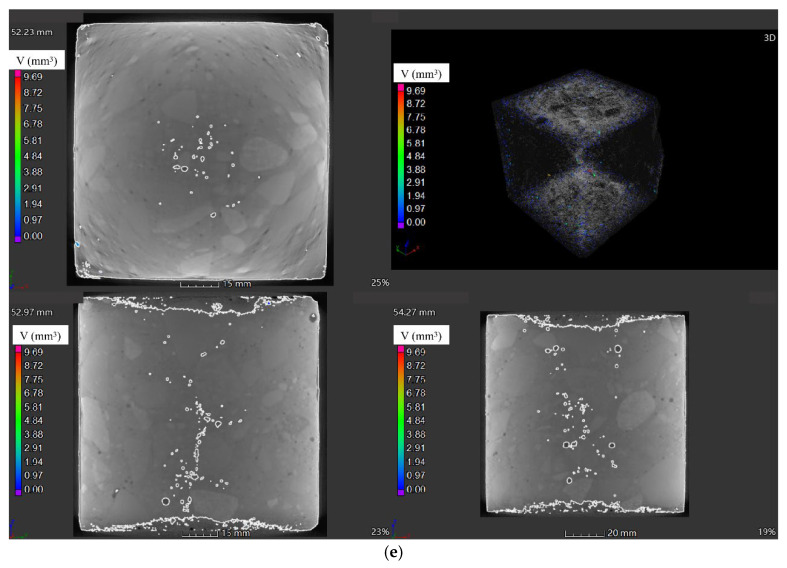
CT of geopolymer concrete. (**a**) B100, (**b**) BF15, (**c**) BF25, (**d**) BS3, (**e**) BS5.

X-ray CT has been widely used as a non-destructive method to characterize the three-dimensional pore structure, internal cracks, and defect connectivity of cement-based materials [32]. Previous studies have shown that CT-based pore structure analysis can effectively reveal changes in porosity, pore distribution, and internal damage evolution under freeze–thaw or salt-freezing environments [33]. Therefore, in this study, CT was used to quantify pore volume and porosity and to further explain the internal damage evolution of FA/SF-modified geopolymer concrete. Figure 20 shows the three-dimensional CT reconstruction images and quantitative pore structure results of geopolymer concrete with different FA and SF dosages. The CT quantitative analysis showed that the pore volumes of B100, BF15, BF25, BS3, and BS5 were 516.3, 925.76, 274.61, 641.81, and 392.9 mm^3^, respectively, and the corresponding porosities were 0.0516%, 0.0926%, 0.0275%, 0.0642%, and 0.0393%, respectively. Compared with the control group B100, the porosity of BF15 increased, indicating that the incorporation of FA may increase internal defects and pore volume. However, the porosity of BF25 was lower than that of BF15, which may be related to the filling effect of FA particles and the local heterogeneity of CT sampling. For the SF groups, the porosity of BS3 was slightly higher than that of B100, whereas BS5 showed a lower porosity than BS3. This indicates that SF can refine the pore structure to some extent, but the pore evolution is affected by both the filling effect and the local distribution of reaction products. Overall, the CT quantitative results provide direct pore volume and porosity data, which further support the discussion of internal defects and microstructural changes in different specimens. When 15% FA was incorporated, the number of small pores increased significantly. FA was able to fill the interior of the concrete, which reduced the pore size. After excessive FA was incorporated, the cement clinker was overly diluted. The formation of hydration products was insufficient. The originally isolated fine pores gradually connected with each other. This formed a connected pore network. This indicated that a high FA content led to a loose matrix and the formation of a large number of fine pores. For the SF groups, the BS3 group exhibited the best pore structure refinement effect. The pore distribution images showed a significant reduction in the number of pores. The pores were mainly isolated small pores. The porosity of the BS5 group increased compared with that of the BS3 group. Signs of some connected pores also appeared. This indicated that when the SF content was increased to 5%, it still had a certain filling effect. However, compared with the BS3 group, the BS5 group showed more pores and local defects in the CT results, which may explain its weakened improvement effect. The changes in the CT pore structure originated from the low activity and slow hydration of FA. Excessive FA incorporation led to a loose matrix and increased porosity. In contrast, SF had high activity. An appropriate amount (3%) could fill pores and optimize the structure. However, an excessive amount (5%) increased defects due to agglomeration. These results were consistent with the macroscopic performance and SEM observations. This confirmed that an appropriate amount of SF was an effective way to optimize the pore structure.

## 4. Conclusions

This study used low-carbon cement as a cementitious material. It systematically investigated the effects of different dosages of FA (15% and 25%) and SF (3% and 5%) on the mechanical properties, durability performance, and microstructure of geopolymer concrete. The concrete was subjected to freeze–thaw cycles and single-sided salt freeze–thaw erosion environments. The main conclusions were as follows:

(1) Compressive strength: The addition of FA significantly reduced the 56 d compressive strength of concrete. The compressive strengths of the BF15 and BF25 groups decreased by 10.9% and 13.02%, respectively, compared with the control group. A higher FA content led to lower strength. In contrast, the addition of SF significantly increased the compressive strength. The BS3 group showed the best improvement effect, with an increase of 13.24% compared with the control group. The BS5 group showed an increase of 9.15%, which was slightly lower than that of the BS3 group.
(2) Freeze–thaw resistance: As the number of freeze–thaw cycles increased, the compressive strength, mass loss, and relative dynamic elastic modulus of all specimens deteriorated. A higher FA content led to more significant deterioration in freeze–thaw resistance. After 100 freeze–thaw cycles, the compressive strength of the BF25 group decreased by 48.3% compared with the control group. Its mass loss reached 2.84%. Its relative dynamic elastic modulus dropped to 64.86%. The BS3 group (3% SF) exhibited the best freeze–thaw resistance. After 100 freeze–thaw cycles, its relative dynamic elastic modulus remained at 86.64%. Its mass loss was only 0.72%. When the SF content was increased to 5%, the improvement effect weakened. The freeze–thaw resistance of the BS5 group was lower than that of the BS3 group.
(3) Salt-frost scaling resistance: As the chloride ion concentration of the salt solution increased, the splitting tensile strength of all concrete groups gradually decreased. The surface scaling mass increased. The BS3 group maintained the lowest scaling mass under all salt concentrations and freeze–thaw cycle numbers. It exhibited the best salt-frost scaling resistance. A high FA content (25%) significantly aggravated salt-frost scaling. After 100 single-side salt freeze–thaw cycles at a salt concentration of 7%, the scaling mass of the BF25 group reached 1130 g/m^2^. This was much higher than that of the control group (640 g/m^2^). From an engineering perspective, the BS3 mixture showed the best overall resistance to freeze–thaw damage, salt-frost scaling, and chloride ingress, indicating its potential suitability for geopolymer concrete structures exposed to cold coastal environments. However, the use of high FA content should be carefully evaluated in such environments because it may increase pore connectivity and chloride transport risk.
(4) Microstructural mechanisms: SEM-EDS analysis indicated that an appropriate amount of SF (3%) significantly increased the Si/Al ratio and C-S-H gel content. It also densified the matrix. In contrast, excessive FA (25%) led to a sharp decrease in the Ca/Si ratio. It also increased the number of unhydrated particles and promoted microcrack propagation. XRD analysis confirmed that the incorporation of SF promoted the formation of C-S-H gel and ettringite. In contrast, the incorporation of FA diluted the cement clinker and reduced the amount of hydration products. CT analysis further showed that the BS3 group had the optimal pore structure, which was dominated by isolated small pores. In contrast, the BF25 group had the highest porosity and formed a connected pore network.

### Future Perspectives

Future perspectives: Although this study investigated the durability and microstructure of FA/SF-modified geopolymer concrete under freeze–thaw and single-side salt-freezing conditions, several limitations remain. First, SEM-EDS and XRD provided indirect evidence for the formation of C-(A)-S-H/N-A-S-H-related inorganic gel products, but the exact Si–O–Al bonding environment and gel polymerization degree were not directly determined. Future studies should include FTIR, TG-DTG, and NMR to further characterize the inorganic polymer gel network. Second, the CT analysis quantified pore volume and porosity, but more detailed parameters such as pore size distribution, crack connectivity, and tortuosity should be further analyzed using image segmentation methods. Third, fresh properties such as slump, flowability, air content, and rheological behavior were not measured, and their influence on SF dispersion and pore formation requires further investigation. Finally, only selected FA and SF dosages were studied, and long-term exposure tests under real coastal environments are needed to verify the engineering applicability of the proposed mixtures.

## Figures and Tables

**Table 2:** Chemical composition of raw materials, FA, and SF (wt%).

Samples	SiO_2_	K_2_O	MgO	Na_2_O	Fe_2_O_3_	SO_3_	Al_2_O_3_	CaO	Others
B	28.42	0.452	7.95	0.838	0.703	12.56	18.06	29.93	1.087
FA	50.25	1.67	3.53	4.57	4.12	1.2	26.05	7.19	1.42
SF	86.71	3.3	3.19	2.36	1.3	0.821	0.778	0.753	0.788

**Table 3:** Proportion of low-carbon concrete.

Number	B (kg/m^3^)	FA (kg/m^3^)	SF (kg/m^3^)	Sand (kg/m^3^)	Recycled Coarse Aggregate (kg/m^3^)	Water (kg/m^3^)	Water-Reducing Admixture (kg/m^3^)
B100	500	0	0	678	1106	150	2
BF15	425	75	0	678	1106	150	2
BF25	375	125	0	678	1106	150	2
BS3	485	0	15	678	1106	150	2
BS5	475	0	25	678	1106	150	2

Note: B100 represents cement; BF refers to the addition of FA; BS refers to the addition of SF. For example, BF-25 indicates that 25% of the cement is replaced by FA, and BS-5 indicates that 5% of the cement is replaced by SF.

**Table 4:** Depth of erosion.

Layer	1	2	3	4	5
Distance between the center of the sample and the surface (mm)	2.5	12.5	22.5	32.5	42.5

## Data Availability

The original contributions presented in this study are included in the article. Further inquiries can be directed to the corresponding author.
